# Stages of treatment of eating disorders in endogenous depressions

**DOI:** 10.1192/j.eurpsy.2023.1105

**Published:** 2023-07-19

**Authors:** A. Barkhatova, A. Smolnikova, M. Bolgov

**Affiliations:** Department of endogenous mental disorders and affective states, Mental health research center, Moscow, Russian Federation

## Abstract

**Introduction:**

The problem of eating disorders has become increasingly important in recent years, due to the increase in the number of cases among children and adolescents, as well as the insufficient effectiveness of therapeutic measures. An important role in the course of eating disorders is occupied by the depressive syndrome associated with it, which complicates the process of treatment and rehabilitation in this pathology.

**Objectives:**

Study of the stages of treatment of eating disorders in the structure of depressive states.

**Methods:**

The sample consisted of 63 patients aged from 15 to 25 years old (all female, average age 16.2), who were on outpatient and inpatient observation in the clinic were studied.

**Results:**

In the process of treatment, several stages of treatment of patients were carried out. The first stage was aimed at normalizing the body’s vital functions and management of somatoendocrine impairments (the duration of the stage is about 14 days). The next stage was aimed at the psychotropic treatment of eating disorders and concomitant mental pathologies (the duration of the stage is 3-4 weeks). The final stage included rehabilitation, which consisted of working with a psychotherapist (the duration of the stage was 8 weeks or more). It should be noted that in the process of rehabilitation, patients continued to receive psychopharmacotherapy and underwent a comprehensive examination to assess the dynamics of their condition.

**Conclusions:**

Eating disorders in the structure of endogenous depressions require an integrated approach to treatment, including both ensuring adequate vital activity of the organism and the selection of drug treatment depending on the nosological affiliation of the underlying syndrome. Rehabilitation work aimed at social adaptation and prevention of relapses of the disease also plays an important role.

**Disclosure of Interest:**

None Declared

